# A Multilevel Analysis of Associations Between Children’s Coloured Progressive Matrices Performances and Self-Rated Personality: Class-Average and Class-Homogeneity Differences in Nonverbal Intelligence Matter

**DOI:** 10.3390/jintelligence13080095

**Published:** 2025-07-30

**Authors:** Lisa Di Blas, Giacomo De Osti

**Affiliations:** Department of Life Sciences, University of Trieste, 34127 Trieste, Italy; giacomo.deosti@studenti.units.it

**Keywords:** nonverbal intelligence, self-rated personality, elementary school students, class-homogeneity, multilevel modelling

## Abstract

The relationship between self-rated personality and nonverbal intelligence has been studied in young students, but these studies have generally not considered nested data, despite their allowing us to analyse between-classroom variability. The present cross-sectional study involved third- to sixth-grade students (n = 447) who were nested into their classrooms (n = 32). The participants completed the Raven’s Coloured Progressive Matrices (CPM) as a measure of nonverbal intelligence and a personality questionnaire based on the Five Factor Model. At the class level, the study data included class size, class-average CPM scores, and class-homogeneity in CPM performances. Multilevel modelling with class-mean centring of personality predictors was applied to examine class-average differences in CPM scores and interaction effects between personality and class-homogeneity on CPM scores. The results showed significant differences in average CPM performances across classrooms, significant fixed and random slope effects linking nonverbal intelligence and Imagination, and a cross-level effect revealing that Imagination is a stronger predictor of CPM scores when class-homogeneity in intelligence is lower. Beyond confirming the intelligence–Imagination association generally observed in the literature, the present findings emphasise the importance of using nested structures when collecting personality and intelligence data in classrooms. More attention needs to be paid to how the classroom environment affects children’s self-reported personality and intelligence test performances.

## 1. Introduction

Personality and intelligence are two fundamental psychological constructs that have been developed to describe and understand individuals and their underlying functioning. The so-called Five Factor Model (FFM) is currently a reference framework that hierarchically systematizes the most relevant individual differences into five higher-ordered domains, namely, Extraversion, Agreeableness (Benevolence in childhood), Conscientiousness, Emotional Stability, and Openness to Experience (Imagination in childhood) (McCrae and John 1992; Mervielde et al. 2009). This model has been extensively tested from biological, cross-cultural, developmental, practical, and clinical perspectives (Costa et al. 2019; Jang et al. 1996; McCrae and Sutin 2018; Mervielde and De Fruyt 2000; Widiger and Crego 2019). Intelligence is also a complex, biologically based (Deary et al. 2022; Neubauer and Fink 2009) and hierarchically structured human domain. It underlies an individual’s performance and adaptive abilities when dealing with and shaping their environment (Sternberg 2012). Traditionally, a general intelligence factor includes crystallised or learning abilities, which tend to increase across the lifespan; nonverbal or fluid intelligence, which primarily represents problem-solving and reasoning abilities and reaches a peak in early adulthood (Anglim et al. 2022); and additional visuospatial capabilities and speed (Lang et al. 2016).

Researchers have long been working to understand how intelligence and personality connect to each other and interact. Theoretically, correlations between intelligence and the FFM domains are expected. Following Cattel’s investment theory, which was further developed in Ackerman’s theory of intelligence as Process, Personality, Interests, and Intelligence-as-Knowledge (PPIK; von Stumm and Ackerman 2013), higher Openness to Experience is expected to favour an individual’s greater time and effort investment in their fluid intellectual development, which strengthens crystallised intelligence over the years (Trapp et al. 2019), fostering, in turn, a greater enjoyment of intellectual challenges and achievements. Extending the investment theory to Conscientiousness, i.e., being systematic and being persistent toward an objective, both additive and synergistic effects of intelligence and Conscientiousness have been hypothesised, to predict better performance (Brandt and Lechner 2022). Alternatively, Conscientiousness may compensate for lower intelligence, and thus also enable a considerable level of performance (Moutafi et al. 2006).

In general, a negative correlation between intelligence test performance and Emotional Instability is expected; anxiety impairs processing efficiency and therefore requires increasing efforts to improve the quality of the outcome (Eysenck and Calvo 1992; Moutafi et al. 2005). Nevertheless, the intelligence–Neuroticism relationship is more complex (Schermer et al. 2024). For example, the deficit model suggests that lower intelligence anticipates higher test anxiety (Sommer and Arendasy 2014). It has also been reported that the intelligence–Neuroticism link could be negative in healthy people but positive in patients suffering from generalised anxiety disorders (Moghadasin and Dibajnia 2021).

Regarding the interpersonal FFM domains, higher intelligence underlies a skilful processing of social information and protects against aggressiveness, peer rejection, and higher externalizing and risk behaviour problems, such as excessive alcohol and drug use, in early and middle adolescence (Dodge and Crick 1990; Huepe et al. 2011). Finally, introversion has been reasoned to favour interests in general learning rather than in social experiences (Schmidt 2014), leading to a negative Extraversion–intelligence association. Developmentally, the maturation hypothesis suggests a shift from a positive to a negative correlation in middle adolescence, but studies have yielded mixed and negligible correlations (*ρ* = −0.04; Wolf and Ackerman 2005).

The empirical results of a recent and representative meta-analytic study (Anglim et al. 2022) indicate that both crystallised and fluid intelligence correlate with Openness (*ρ* = 0.20), with the facets of Intellectual Curiosity, Ideas, and Values making a significant positive contribution ($\bar{r}>0.20)$, and with Emotional Instability making a significant negative contribution (*ρ* = −0.09). Over the course of life, personality–intelligence associations do not change, meaning that when attention is focused on late childhood, the results generally mirror findings from adult samples. Nevertheless, children’s personalities are often assessed through their parents’ or teachers’ reports, and results suggest that lower Extraversion, higher Imagination, and higher Conscientiousness are positively associated with nonverbal intelligence, with Concentration and Perseverance facets contributing positively, but Orderliness contributing negatively (Di Blas and Carraro 2011; Moutafi et al. 2006). In addition, interaction effects between a child’s personality and a parent’s gender suggest that mothers, but not fathers, attribute higher Conscientiousness to their more capable sons (Di Blas and Carraro 2011).

Fewer findings on personality–intelligence are available wherein children’s self-reports of their personality are inspected. Recent empirical studies on the investment theory of intelligence and Conscientiousness analysed the hypothesis that fluid intelligence and self-reported Conscientiousness interact to yield greater improvements in math and reading two years later in fourth- and in seventh-graders (Brandt and Lechner 2022). These results showed that Conscientiousness and fluid intelligence were not associated concurrently, but both contributed additively (not synergistically) to competence improvements across time. Bardach et al. (2023) conducted a longitudinal study on school students, aged between 11 to 14 years, and found that lower self-rated Extraversion and Conscientiousness scores predicted later decreases in intelligence test performances; no associations emerged for the remaining FFM scales across time. Finally, Johann and Karbach (2022) studied fourth graders and found a negative association between fluid intelligence via Raven’s matrices scores and Emotional Stability, controlling for working memory, inhibition of interference, and flexibility costs; conversely, a positive correlation between fluid intelligence and Conscientiousness was observed in their sample of emerging adults.

Schooling significantly influences both fluid and crystallised intelligence, beyond socioeconomic status or genetics, across the lifespan (Judd et al. 2022; Ritchie and Tucker-Drob 2018). During their school years, children spend much of their time in the classroom, with their classmates and teachers. The classroom represents a fundamental learning and developmental environment that shapes, for example, a student’s math anxiety (O’Hara et al. 2022). Despite this, a large majority of studies on school students treat and analyse data under the assumption that they are randomly sampled, thus disregarding the fact that the students are nested into classrooms and that between-classroom variance, i.e., class-average differences in intelligence test performances, should be considered in the analytical models. Indeed, some studies have hypothesised and demonstrated class-average differences in children’s emotional and behavioural problems from primary to secondary school years (Wang et al. 2018) and in the accuracy of teachers’ judgment of their students’ intelligence (Baudson et al. 2016). In addition, from an educational perspective, ability heterogeneity in the classroom is also a relevant factor. In fact, to how to treat mixed-ability classrooms is still under debate, i.e., whether within-class ability grouping allows students to advance at their own rate or whether it creates differential learning experiences and expectancies that impair some students in the long run (Du Plooy 2019). Conversely, to our knowledge, no attention has been paid to class-average and class-homogeneity differences in intelligence test scores and to their effects on personality–intelligence associations.

### Study Aims and Hypotheses

The main aim of the present cross-sectional study was to investigate the associations between self-rated personality and nonverbal intelligence in children between the third and sixth grades. Specifically, we adopted a mixed modelling approach, with students nested into classrooms, and tested the following hypotheses: (*H1*) There are significant differences in average levels of nonverbal intelligence across classrooms. (*H2*) The associations between children’s self-rated personality and nonverbal intelligence, at the within-classroom level, parallel the relationships between personality and fluid intelligence generally reported in the literature, with Imagination being positively associated and Emotional Instability being negatively associated with intelligence, whereas no association direction was predicted for Conscientiousness and Extraversion (Anglim et al. 2022; Asendorpf and Van Aken 2003; Moutafi et al. 2006; Wolf and Ackerman 2005). Mixed modelling allowed for the further exploration of whether personality—intelligence associations vary across classrooms. (*H3*) Personality domains interact with each other in accounting for nonverbal intelligence, i.e., Imagination is expected to synergistically interact with Introversion (Schmidt 2014) and Conscientiousness (Brandt and Lechner 2022), whereas an antagonistic moderating effect of Emotional Instability is hypothesised (Eysenck and Calvo 1992). (*H4*) The degree of class homogeneity in intelligence interacts with personality, i.e., personality domains are stronger predictors of individual differences in nonverbal intelligence when within-class variability or heterogeneity in intellectual abilities is higher.

## 2. Materials and Methods

### 2.1. The Study Sample

On the whole, 509 children (236 males, 46.3%) took part in this study; 139 (61 males) were third graders, 143 (72 males) were fourth graders, 146 (72 males) were fifth graders, and 81 (32 males) were sixth graders. The students represented the level 1 sample, and they were nested into their classrooms (i.e., 32 classes), or the level 2 sample, with class size ranging from 9 to 22 (median = 17).

### 2.2. Procedure

All the children were recruited from public schools. By law (DPR, 81, 2009), in Italy, children are assigned to classrooms following a between-classroom criterium of equal distribution and a within-classroom criterium of heterogeneity in terms of gender, religion, and nationality. No ability or behavioural tests are allowed to be applied to children to distribute them across classrooms. Children with certified cognitive disabilities or behavioural and emotional difficulties are assigned to different classrooms following the criterium of equal distribution. Furthermore, in the present study, children (i.e., classes) from several schools in different areas of Northeastern Italy were involved to strengthen sample representativeness.

The children took part in the study after their parents agreed and submitted a written, individually signed consent form allowing their children to participate. We were not allowed to ask for family cultural background or SES. The children were Italian speakers, capable of understanding and responding to questionnaires independently. They were reassured that they could take part in the study on a voluntary basis only, that they had no time limits, that the questionnaires were anonymous, and that they could withdraw at any time. Accordingly, they were asked to provide no personal sociodemographic variables. Girls and boys were given personality questionnaires with different colours. Each child placed their materials in an envelope to ensure complete anonymity and privacy.

Trained graduating students collected data in the classrooms in a paper-and-pencil format. Data were collected in spring (March to May) in the years 2018 and 2019. These data have not yet been published or shared even partially.

### 2.3. Measures

#### 2.3.1. Raven’s Coloured Progressive Matrices (CPM, Raven 2000)

Children’s nonverbal intelligence was tested by the CPM test, which assesses a 5-to-12-year-old child’s ability to make meaning out of confusion and determine logical links between abstract figures, thus handling complexity (Raven 2000). CPM test scores were calculated by summing all 36 CPM items, and 493 children completed the CPM test (16 pupils provided a largely incomplete response sheet). The present CPM raw scores correlated with school grade at r = 0.30 (*p* < .001) and were standardised by school grade, further transforming the z-scores into IQ scores with M = 100 ± 15. The students with a CPM score equal to or greater than 70 numbered 474 (96.1%).

#### 2.3.2. Five Factor Model Scales for Children

The *Interpersonal Behaviours Questionnaire for Children* (IBQ-C, Di Blas et al. 2012) was administered to evaluate children’s self-views with respect to the interpersonal domains of Extraversion and Benevolence (Mervielde et al. 2009). Specifically, the IBQ-C questionnaire was developed for 8-to-11-year-old children and includes 48 behaviour-descriptive items, which validly indicate how a child perceives their typical way of acting towards other children (4-point Likert scales ranging from 1 = *never* to 4 = *several times*). Developed following the interpersonal circumplex model, the main IBQ-C interpersonal domains of Dominance and Love are rotational variants of the FFM Extraversion and Benevolence domains (Di Blas et al. 2012). In the current study, we calculated IBQ-C scores as rotational variants of FFM Extraversion (investing energy in organizing games, being sociable, and affirming personal competences) and FFM Benevolence (being supportive and warm, respecting rules, and avoiding conflicts).

The non-interpersonal FFM scales of Conscientiousness (staying concentrated, being persistent, and tidy) Emotional Instability (feeling easily scared, worried, sad, discouraged), and Imagination (loving reading and learning new things, having a good memory, having a lot of imagination) were initially assessed by generating 10 short behaviour-descriptive items for each scale, based on the Italian validated version of the HiPIC Hierarchical Personality Inventory for Children (De Fruyt et al. 2014) and the Big Five Questionnaire for Children (Barbaranelli et al. 2003). The structural validity of these 30 items was examined preliminarily. Initially, 488 children completed the IBQ-C and the non-interpersonal scales, and among them, 463 had an IQ greater or equal to 70; overall, missing values were 0.0035% and were replaced with the item average value.

#### 2.3.3. Classroom Variables

Class size (M = 16.9 ± 3.4), class average CPM scores, and class homogeneity as within-class standard deviation of intelligence test scores were examined.

### 2.4. Analyses

Preliminarily, we tested the latent factor structure of the 30 items aimed at assessing the FFM domains of Conscientiousness, Emotional Instability, and Imagination. We applied SEM analysis (Jamovi 2.6.23.0, module semlj-SEM 1.2.4) with Weighted Least Squares Means and Variance adjusted (WLSMV) estimation method for ordinary data, and used the following quantitative fit indices to statistically test how well the hypothesised structural model aligned with the study’s empirical data (Hu and Bentler 1999): Comparative Fit Index (CFI, with a cutoff value close to 0.95 and ≥0.90), Tucker–Lewis Index (TLI, with s cutoff value close to 0.95 and ≥0.90), Standardised Root Mean Square Residual (SRMR, with a cutoff value of ≤0.08 for an acceptable fit), and Root Mean Square Error of Approximation (RMSEA, with a cutoff value close to 0.05 indicating a good fit, and a cutoff value close to ≤0.08 indicating an acceptable fit), with the *p*-value for RMSEA indicating the probability that its value is statistically equal to a RMSEA value of 0.05.

Simple correlation and regression analyses were first applied following a random sampling approach at the individual level. Multilevel modelling was then applied, nesting students into their classroom, to examine within-classroom associations between intelligence and personality, class-average differences in CPM performances, and cross-level (i.e., individual by classroom) interactions. Each student’s self-rated personality scores were centred around the mean of their classroom, with the centred scores representing an individual level compared to that of their classmates (level 1 variable), rather than to that of the overall sample. Differently from grand-mean centring, with each student compared to all the other students, regardless of their classroom, group- or class-mean centring allows for the disentangling of the level 1 (student-level) and level 2 (class-level) effects on interindividual differences and their associations. Class-mean centring should be applied when the research questions examine whether the personality–intelligence relationship varies across classrooms—i.e., if a slope linking a personality domain with Raven’s matrices scores has a random effect, in addition to a fixed effect—and whether level 1 predictors interact in predicting the outcome to determine an unbiased estimation of the associations (Peugh 2010).

Class size, class average, and class homogeneity of CPM scores in the classroom were level 2 variables.

We conducted data analysis via Jamovi 2.6.23.0, module GAMLj3 (version 3.4.2), and applied the default command of the restricted maximum likelihood (REML) estimate method. Random intercept and predictor effects were not allowed to covariate, and random effects for independent level 1 variables were added to the model if statistically significant.

## 3. Results

### 3.1. Preliminary Confirmatory Factor Analysis of the Non-Interpersonal Personality Domains

We analysed the factor structure of the items designed to assess the non-interpersonal domains of Conscientiousness, Emotional Instability, and Imagination, and initial results indicated that some items were weak indicators; therefore, eight items for each domain were retained and submitted to CFA again.

The scaled fit indices, i.e., indices adjusted to account for non-normality in the data, showed an acceptable fit (RMSEA = 0.056, 95% CI 0.050–0.062, *p* = 0.043; SRMR = 0.071; CFI = 0.96; TLI = 0.93), and robust fit indices were weaker but still acceptable (RMSEA = 0.077, 95% CI 0.070–0.085, *p* < .001; SRMR = 0.063; CFI = 0.83; TLI = 0.82). Figure S1 illustrates the factor structure of the items. For each of the scales, responses were averaged across the selected eight items and transformed into z-scores by gender.

Successive analyses included children with Raven’s CPM scores equal to or greater than 70 (range between 70 and 119 IQ scores) and with personality scores greater than or equal to −3.0 z-scores (range between −2.84 and 2.62 z-scores); thus, the impact of outliers was minimised, and both between- and within-class variability were not artificially inflated. Overall, the results presented herein were based on a sample of n = 447, i.e., 93.7% of the respondents to both intelligence and personality instruments.

### 3.2. Descriptive Statistics and Simple Correlations Between the Study Variables

Table 1 presents the internal consistency coefficients of the study variables and the bivariate simple correlations between them. Internal consistency levels are adequate. The results show a significant correlation between standardised CPM scores and self-rated Imagination. When multiple regression analysis was applied, with the five personality estimators entered simultaneously in the model, the regression model accounted for R^2^ = 0.046 (*p* > 0.001) and the significant estimators were Conscientiousness (β = −0.17, *p* = 0.006) and Imagination (β = 0.25, *p* < 0.001). Emotional Instability moderated the impact of Imagination (β = −0.09, *p* < 0.05, ΔR^2^ = 0.008) on IQ performances antagonistically, i.e., weakening the positive link between Imagination and CPM scores.

Table 1 also shows how the FFM personality domains are intercorrelated; correlations are in line with the literature (De Fruyt et al. 2014; Mervielde et al. 2009).

### 3.3. Intra-Class Correlation (ICC) Coefficients (H1)

The random intercept model was applied to the dataset to inspect how class membership accounts for variance in the study variables of both intelligence and personality. The results (Table 2) show class-average differences, i.e., pupils studying within the same classroom tend to have more similar intelligence, Conscientiousness, and (marginally) Benevolence levels to one another than children in other classrooms. The results in Table 2 indicate that class membership accounts for a substantial variance proportion of CPM scores and supports *H1*, further showing that class membership accounts for differences in being diligent and persistent and, marginally, in being kind and helpful. Therefore, a mixed modelling analytic approach must be applied to the current data instead of a traditional between-people correlational approach.

### 3.4. Within-Class Associations Between Children’s Intelligence and Self-Reported Personality (H2, H3)

Table 3 shows the results from a random intercept and random slope model after class-mean centring. Model (*H2*) shows a within-class association between self-reported Imagination and CPM scores (*p* < 0.001), further revealing a random slope effect for Imagination; that is, the results indicate that the association between the predictor and the outcome varies in intensity across classrooms. Conscientiousness was marginally significant (*p* = 0.069). The fixed effects of the personality variables accounted for 3.2% of the outcome variance. No significant interaction effects (*H3*) emerged. Overall, these results indicate that not only are class-average differences in IQ levels relevant, but class differences in Imagination–nonverbal intelligence associations are also relevant.

Table 3 also presents the results from mixed modelling after centring variables around their grand mean, i.e., disregarding that students are nested into classrooms when associations between predictors and outcome are inspected. The results reflect those observed in the regression analysis, with Conscientiousness also being significant and negatively associated with CPM scores, with an increase of 1 SD in self-reported Conscientiousness predicting a decrease of 1.80 IQ (i.e., in the present dataset, approximately −0.16 z-scores in CMP performance for each +1 z-score in Conscientiousness); moreover, when interaction effects were added to the model, Imagination by Emotional Stability was also statistically significant (b = −1.13, *p* = 0.05), indicating that lower Emotional Stability levels weakened the positive association between Imagination and CPM scores.

Imagination was inspected as a dependent variable, from a class-mean centring approach, and the results indicated that CPM scores were significantly associated with Imagination (*p* < 0.001), in addition to the personality variables of Extraversion (*p* = 0.033), Benevolence (*p* < 0.002), and Conscientiousness (*p* < 0.001).

### 3.5. Class Homogeneity in Intelligence Moderates Personality–Intelligence Associations (H4)

According to *H4*, not only should the variability in intelligence mean levels across classes be considered (*H1*); the variability in the homogeneity of CPM scores in classes should also be inspected. We calculated the within-class homogeneity of CPM scores as the class standard deviation of scores around the class mean (n = 447, class n = 32); i.e., the lower the level, the higher the class homogeneity (M = 11.02 ± 2.56). Table 4 presents the results before and after the cross-level interaction between class homogeneity and Imagination (no other personality factors interacted significantly) was added to the model. Compared to the model class-mean centring in Table 3, Model 1 in Table 4 adds the level 2 predictors of class homogeneity and class size and shows that expected average nonverbal intelligence is higher when class homogeneity is higher (i.e., +1.77 IQ if within-class SD = −1 in our dataset). Class size was not a significant predictor. Model 2 in Table 4 adds the cross-level effect between self-rated Imagination and class homogeneity. The results indicate that Imagination is a stronger predictor when class homogeneity is lower, in line with *H4*. Marginal ΔR^2^ is 0.026 (*p* < .001). This suggests that the interaction effects account for an additional non-negligible variance proportion. The random slope effect for Imagination was no longer significant.

## 4. Discussion

The present cross-sectional study examined how nonverbal intelligence, assessed via Raven’s Coloured Progressive Matrices, and self-rated personality, as FFM domains, are associated in late childhood. In contrast to previous research studies conducted in school populations, the present study adopted a multilevel approach and nested the young participants into their classrooms. Such an approach revealed that class-average nonverbal intelligence varies substantially; conversely., class size has no effect on individual differences in nonverbal intelligence. Rix and Ingham (2021) underlined that pupils are often assigned to their classrooms non-randomly, but taking into account prior information about children’s capabilities and behaviours. In the present study, we collected data in public schools, where, by law, criteria favour a uniform distribution of individual differences across classrooms and heterogeneity within classrooms. Nevertheless, we found substantial differences. Such a finding is of relevance to educational and developmental psychology, but it needs to be further investigated in order to be understood. In fact, we did not systematically inspect possible underlying variables accounting for such differences, such as SES background, which significantly affects individual differences in intelligence and intelligence growth from ages 2 to 16 (von Stumm and Plomin 2015), or classroom climate the teachers significantly influence, which also shapes the students over time (Baudson et al. 2016; Erdem and Kaya 2024; O’Hara et al. 2022), thus favouring the development of cognitive abilities differently both within and between classrooms. A longitudinal study could reveal if differences in intelligence performance across classrooms are present since the first elementary school year or if they develop in the later years.

Within-classroom associations between personality and nonverbal intelligence confirmed that Imagination, i.e., enjoying learning new things, loving fantasy, and being a good student, predicts higher intelligence, with an effect size in the range of the traditional literature (Anglim et al. 2022). Our findings also confirm that Conscientiousness and Neuroticism are inconsistent predictors. In addition, our study shows that Imagination emerges as a significant predictor of nonverbal intelligence outcome from both nested and non-nested data into classrooms. Conversely, Conscientiousness was negatively related to nonverbal intelligence only when non-nested data were examined.

The present results confirm that Imagination and Conscientiousness do not interact in the prediction of intelligence test performance (Brandt and Lechner 2022), regardless of whether we applied a multilevel approach or not. Conversely, Emotional Instability antagonistically moderated the positive Imagination–intelligence relation, but only when individual differences were not nested into classrooms. Although such an interaction effect has generally been untested, and although it was small in the present data, it is consistent with the hypothesis that anxiety impairs processing efficiency (Eysenck and Calvo 1992) and underlines that Neuroticism plays a role in understanding the personality–intelligence association (Schermer et al. 2024), even though a direct link cannot be systematically identified (Johann and Karbach 2022). Mostly, the present findings generally confirm that non-nested vs. nested data approaches do not necessarily yield results in the same direction. Further empirical studies are needed to support such an interaction effect.

The current study is unique in that it demonstrates that not only do average intelligence scores change across classrooms, so do class homogeneity levels, which should be systematically taken into account in future research. In fact, the impact of class homogeneity was significant after controlling for class size, and its overall effect suggests that the higher the class homogeneity, the higher the expected average intelligence level. Furthermore, the lower the class homogeneity, the greater the effect of personality differences on test intelligence outcome. Mathematically, lower sample variability distorts and restricts associations between variables. The current study indicates that a greater class homogeneity attenuates the intelligence–Imagination association. Overall, such findings suggest that it is crucial to understand why some classes are more homogeneous than others, i.e., whether their homogeneity varies as a consequence of teaching styles and capabilities, or because of the preselection of students assigned to a particular classroom. Developmentally, such a finding stimulates a systematic longitudinal analysis of dynamics in classrooms among peers, which could reinforce or undermine the development of intellectual capabilities (Premo et al. 2022; Wang et al. 2014).

From a personality psychology perspective, the present results confirm that 8-to-11-year-old children are valid informants on themselves, with self-evaluated Imagination being positively associated with performance on an objective intelligence test. Remarkably, it reveals that class-average differences in personality variables should also be considered carefully (Wang et al. 2018).

### Limitations

This study has several substantive limitations. We collected cross-sectional data only; longitudinal data could reveal different association patterns (Bardach et al. 2023). We nested individual data into classrooms, but classrooms were not nested into schools; that is, we could not test if class differences in fact reflected differences between schools. In our study, such a limitation partly depends on how public schools are organised in Italy, i.e., the transition from elementary school to middle school (i.e., fifth to sixth grade) implies moving to a different school. We did not include variables such as SES or teaching styles, which could contribute to the understanding of differences in class-average and class-homogeneity intelligence levels (Baudson et al. 2016; O’Hara et al. 2022; Du Plooy 2019; Wang et al. 2018). Non-linear, i.e., quadratic associations, remained untested, and we did not explore whether patterns of intelligence–personality relations change between classes with different average intelligence levels (Ackerman 2018).

We applied a refined measure of the FFM interpersonal domains of Extraversion and Benevolence, but global scales of the non-interpersonal domains were not applied. Although the present results provide evidence for their structural validity, the non-interpersonal domains should be refined to assess the middle-level constructs. Indeed, personality–intelligence associations could be better understood at a middle- rather a the higher-construct level (Di Blas and Carraro 2011; Moutafi et al. 2006). Moreover, the HEXACO personality domains (Ashton et al. 2004), which have also been validated in middle school ages (Sergi et al. 2020), could also be inspected, with Honesty–Humility further contributing to individual differences in intelligence (Fries et al. 2022). Lastly, from a personality perspective, we did not examine the differential personality by intelligence hypothesis (Schermer et al. 2024), i.e., by expanding it from individual- to class-average differences.

## Figures and Tables

**Table 1:** Simple correlations between the study variables.

	Internal Consistency	CPM	Ext	Ben	Con	EmIns	Ima
CPM scores	0.75						
FFM (IBQ-C) Extraversion	0.81	−0.01					
FFM (IBQ-C) Benevolence	0.76	0.05	−0.16 **				
FFM Conscientiousness	0.79	−0.01	−0.02	0.54 **			
FFM Emotional Instability	0.75	0.00	−0.43 **	−0.02	−0.06		
FFM Imagination	0.76	0.17 **	0.09	0.40 **	0.55 **	−0.11	
School grade		0.05	0.07	0.03	0.09	0.02	0.01
Class size		0.11 *	−.12 *	0.04	0.05	0.10	0.06

*Note*. Scores were standardised by gender as z-scores for FFM (IBQ-C) Extraversion and Benevolence (following Di Blas et al. 2012) as well as for the FFM non-interpersonal scales. CPM scores were transformed into standardised IQ scores by school grade (M = 103.0 ± 11.4). Internal consistency, as Cronbach’s alpha, is reported for each personality scale, and it is reported as a simple correlation between even and odd items for Raven’s matrices. * *p* ≤ 0.01; ** *p* ≤ 0.001.

**Table 2:** Intraclass correlation (ICC) estimates for the study variables.

	ICC (n)
CPM nonverbal intelligence	0.072 (*p* = 0.002)
FFM (IBQ-C) Extraversion	0.037 (*p* = 0.098)
FFM (IBQ-C) Benevolence	0.045 (*p* = 0.063)
FFM Conscientiousness	0.073 (*p* = 0.002)
FFM Emotional Instability	0.014 (*p* = 0.552)
FFM Imagination	0.006 (*p* = 0.948)

*Note*. n = 32 school classrooms (level 2); n = 447 students (level 1) CPM = Raven’s Coloured Progressive Matrices. Personality variables were standardised by gender.

**Table 3:** Predicting nonverbal intelligence from children’s self-reports along the Five Factor Model domains: Random intercept and random slope model.

	Class-Mean Centring	Grand-Mean Centring
Intercept	102.4 *** (3.18 ***)	102.5 *** (2.95 **)
Extraversion	−0.45	−0.42
Benevolence	0.26	0.41
Conscientiousness	−1.40	−1.80 *
Emotional Instability	0.32	−0.16
Imagination	2.27 ** (2.57 *)	2.80 ***
*Residuals*	111.29	117.19
*Conditional R* ^2^	0.113 ***	0.109 ***
*Marginal R* ^2^	0.032 ** (*p* = .002)	0.043 ** (*p* = .005)

*Note.* Outcome: CPM = Raven’s Coloured Progressive Matrices. n = 447, school classes = 32. * *p* ≤ 0.05; ** *p* ≤ 0.01; *** *p* ≤ 0.001.

**Table 4:** Predicting nonverbal intelligence from children’s personality and class size and homogeneity: Cross-level interaction effects.

	Model 1	Model 2
*Level 1 (FFM domains)*		
Extraversion	−0.45	−0.50
Benevolence	0.26	0.28
Conscientiousness	−1.40	−1.40
Emotional Instability	−0.32	0.214
Imagination	2.47 ** (2.57 *)	2.49 ***
*Level 2*		
Class size	0.20	0.20
Class-average (low) homogeneity	−0.69 *	−0.69 *
*Cross-level interaction*		
Imagination by Class-average (low) homogeneity		0.66 ***
*Conditional R* ^2^	0.116 ***	0.143 ***
*Marginal R* ^2^	0.066 **	0.093 **

*Note.* Outcome: CPM = Raven’s Coloured Progressive Matrices. The lower the value, the higher the class-average homogeneity. n = 44, school classes = 32. * *p* ≤ 0.05; ** *p* ≤ 0.01; *** *p* ≤ 0.001.

## Data Availability

The dataset is available for research purposes by emailing the corresponding author.

## Supplementary Materials

The following supporting information can be downloaded at https://www.mdpi.com/article/10.3390/jintelligence13080095/s1, Figure S1: Self-ratings of personality: Path diagram of the items indicating the latent factors of Imagination (IMA), Conscientiousness (CON), and Emotional Instability (EmIns) (n = 463).
