# Supplementary material for: A Multilevel Analysis of Associations Between Children’s Coloured Progressive Matrices Performances and Self-Rated Personality: Class-Average and Class-Homogeneity Differences in Nonverbal Intelligence Matter
Source: J Intell. 2025 Jul 30;13(8):95. doi: 10.3390/jintelligence13080095 (PMC12387534; doi:10.3390/jintelligence13080095)
Supplement: Supplementary file 1 [file jintelligence-13-00095-s001.zip › jintelligence-3652035-supplementary.pdf]

Supplement Figure S1.

Self-ratings of personality: Path diagram of the items indicating the latent factors of Imagination (IMA), Conscientiousness (CON) and Emotional Instability (EmIns) (n = 447).

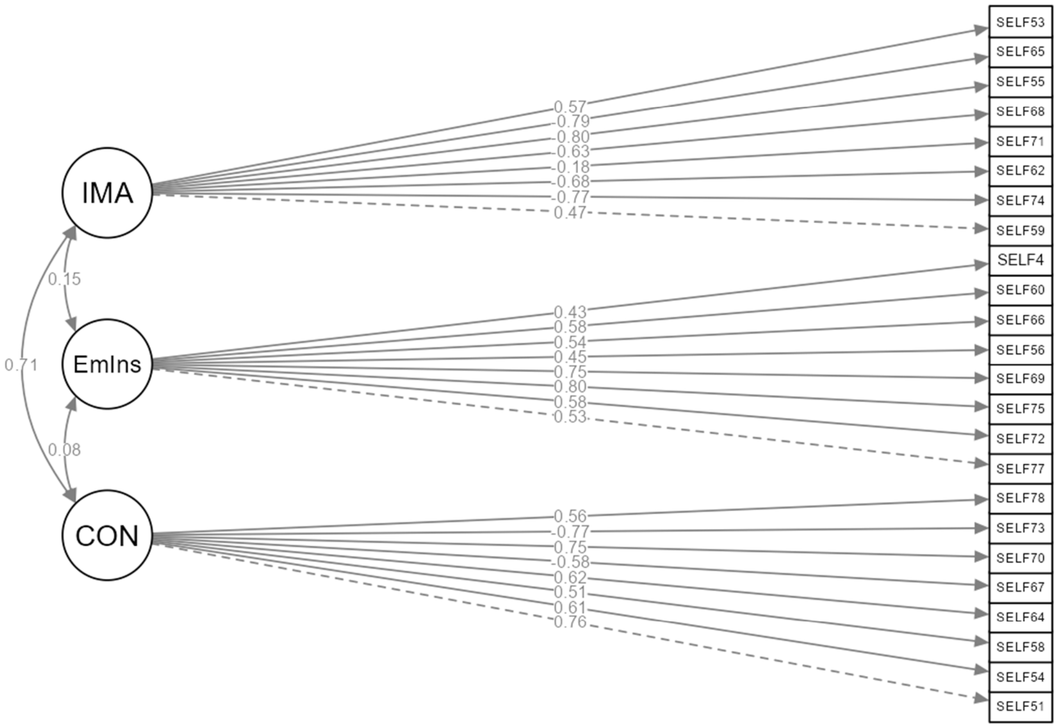

Conscientiousness: 51. During class lessons, I focus on the things I do; 54. When I'm done with my homework, I double-check it to see if I've done everything right; 58. I keep my bedroom tidy; 64. When I start homework, I do my best to finish it, without doing any other things; 67. I am easily distracted in class, when the teacher explains (*to be reversed);* 70. I put a lot of effort into the things I do; 73. I do my homework carefully, without getting distracted; 76. I play after finishing my homework.

Emotional Instability: 49. I think others are better than me; 56. I get scared immediately; 60. I happen to be afraid of not making it, of not being able to do what I would like; 66. I happen to feel sad; 69. When I have to do something, I'm afraid of making mistakes; 72. I worry that something is going wrong; 75. When I want to do something, I worry that I am not capable of doing it; 77. When I see that I am not capable of doing something, I immediately get discouraged.

Imagination: 53. I have a good memory; 55. I easily learn the things I study at school; 59. I read books (apart from those I must read at school); 62. When the teacher asks questions, I know how to answer them well; 65. When the teacher explains something, I immediately understand; 68. I like to learn new things; 71. I have a lot of imagination; 74. When someone explains something to me, I understand immediately.
